# Insights into how Malaysian adults with limited health literacy self‐manage and live with asthma: A Photovoice qualitative study

**DOI:** 10.1111/hex.13360

**Published:** 2021-09-12

**Authors:** Hani Salim, Ingrid Young, Ping Yein Lee, Sazlina Shariff‐Ghazali, Hilary Pinnock, RESPIRE collaboration

**Affiliations:** ^1^ NIHR Global Health Research Unit on Respiratory Health (RESPIRE), Usher Institute The University of Edinburgh Edinburgh UK; ^2^ Department of Family Medicine Universiti Putra Malaysia Serdang Malaysia; ^3^ Centre for Biomedicine, Self and Society, Usher Institute University of Edinburgh Edinburgh UK; ^4^ UM eHealth Unit, Faculty of Medicine University of Malaya Kuala Lumpur Malaysia; ^5^ Malaysian Research Institute on Ageing Universiti Putra Malaysia Serdang Malaysia

**Keywords:** asthma, health literacy, low‐ and‐middle‐income country, Photovoice, qualitative, supported self‐management

## Abstract

**Background:**

Adjusting to life with a chronic condition is challenging, especially for people with limited health literacy, which is associated with low compliance with self‐management activities and poor clinical outcomes.

**Objective:**

We explored how people with limited health literacy understand asthma and undertake self‐management practices.

**Design:**

We adapted the arts‐based qualitative methodology Photovoice.

**Setting and Participants:**

We sampled ethnically diverse adults with asthma and limited health literacy from four primary healthcare clinics in Malaysia. After a semistructured in‐depth interview, a subset of participants took part in the Photovoice component in which participants undertook a 2‐week photo‐taking activity and subsequent photo‐interview. Interviews, conducted in participants' preferred language, were audio‐recorded, transcribed verbatim, translated and analysed thematically. We used the Sorensen's framework (Domains: access, understand, appraise, apply) to describe participants' experience of living with asthma, what they understood about asthma and how they decided on self‐management practices.

**Results:**

Twenty‐six participants provided interviews; eight completed the Photovoice activities. Participants with limited health literacy used various sources to access information about asthma and self‐management. Doctor–patient communication had a pivotal role in helping patients understand asthma. The lack of appraisal skills was significant and experiential knowledge influenced how they applied information. Self‐management decisions were influenced by sociocultural norms/practices, stigmatizing experiences, and available social support.

**Conclusion:**

Locally tailored multilevel interventions (interpersonal, health system, community and policy) will be needed to support people with limited health literacy to live optimally with their asthma in an ethnically diverse population.

**Patient/Public Contribution:**

Patients were involved in the study design, recruitment, analysis and dissemination.

## 1 BACKGROUND

Limited health literacy is a global public health problem that limits people's ability to self‐manage chronic conditions such as asthma.^1^, ^2^ Health literacy is defined as a person's cognitive and functional abilities to respond to the healthcare system's demands to care for their health.^1^ About two‐thirds of people with asthma in a Malaysian population were found to have limited health literacy.^3^ In people with asthma, studies have shown that limited health literacy is associated with increased unscheduled emergency visits and hospitalizations.^4^ Support for self‐management in the form of regular healthcare professional reviews and a written action plan improves asthma outcomes.^5^ As recommended in asthma guidelines,^6^, ^7^ self‐management requires a good understanding of asthma and informed action on the part of the patient. However, asthma self‐management may be challenging to implement and/or support in many health settings.^8^, ^9^ Tailoring self‐management support for people with limited health literacy is crucial but is rarely provided.^10^

Asthma affects almost 360 million globally,^11^ yet it is a neglected noncommunicable disease in many health settings.^12^ In Malaysia, the prevalence of adult asthma was 4.2%, with 1.2% of deaths related to asthma in 2006.^13^ To date, qualitative explorations on asthma and health literacy issues are mainly from high‐income countries.^14^, ^15^, ^16^ A mismatch between patients' and doctors' expectations are among the challenges faced by people with limited health literacy.^14^ Other identified barriers include the doctor's communication style, language and mistrust in the doctor–patient partnership due to perceived racial bias and lack of cultural sensitivity.^14^, ^15^, ^16^ Culture, health beliefs and experiential knowledge influenced self‐management behaviours leading to actions that may not align with evidence‐based practice.^8^, ^14^, ^15^ Individual choices and health decision‐making are governed by deeply embedded sociocultural norms and practices within a community.^2^, ^17^

In this study, we recognized the sociocultural diversity of the population in Malaysia and the need to allow creative ways to explore health literacy, asthma and self‐management practices. Photovoice, an arts‐based qualitative methodology that employs collective conversations,^18^ to understand the issues and strengths of the community and the health system.^19^ We adapted Photovoice by combining qualitative interviews and photo‐taking activities to explore experiences and employ innovative approaches to disseminating the voice of the community. We aimed to explore how people with limited health literacy in Malaysia understand asthma and decide on self‐management practices.

## 2 METHODS

Ethical approval was granted by the Medical Research and Ethics Committee of the Ministry of Health, Malaysia (ID: NMRR‐18‐2113‐42322) and sponsorship approval by the Academic and Clinical Central Office for Research & Development at the University of Edinburgh (ACCORD) (ID: AC18113). Informed consent was obtained before participation.

## 3 STUDY DESIGN

**Figure 1:**
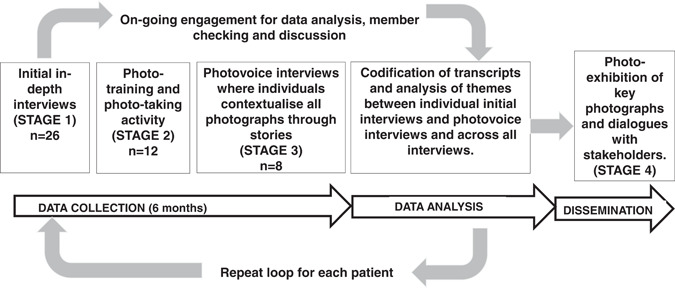
Our adapted Photovoice process

We adapted Photovoice,^18^ an arts‐based qualitative methodology (Figure 1) to generate understandings of health literacy challenges among people with asthma in Malaysia. The study included four stages. Stage 1 involved one‐to‐one in‐depth interviews. Stage 2 involved training and photo‐taking activity. Stage 3 involved one‐to‐one Photovoice discussions with the subset of participants who took photos. A small‐scale exhibition and sharing of findings through local social media networks took place at the end of the study to amplify people's experiences living with asthma among stakeholders (Stage 4). Wang's classic Photovoice description was of a one‐way process where recruited participants underwent training, photo‐taking activities and discussion as a group.^18^ Although participants were given the freedom to take the photographs as individuals, only key photographs were chosen and discussed in focus group discussions.^18^ The first group engagement was to train in Photovoice technique and understand the research assignment followed by group dialogue with policy‐makers using key photographs.^18^ Wang and Burris^18^ completed the Photovoice stage with a final participants' evaluation of the process. We adapted this process to conform to our population's sociocultural needs. We introduced an in‐depth interview in the first stage as a strategy to secure initial engagement, to foster relationships and trust among the research participants. We invited interviewees to take photographs and discussed all their photographs in a confidential interview.

### 3.1 Setting, recruitment and sample

The study was conducted in 2019 at four primary health clinics in Klang District, a central region of Malaysia (see Table 1 for a description of Malaysia's healthcare system). These clinics were chosen to reflect a range of urban and suburban populations enabling the recruitment of a broad range of participants. Pictorial advertisements and easy‐read information about the study were placed at the study sites. H. S. explained the study procedures to potentially interested participants (face‐to‐face or through phone calls) and assessed eligibility. Video information sheets helped potential participants, regardless of reading ability, visualize the research process, understand what was involved and make an informed decision about participation. The video was made available online where participants could watch it in their own time^9^ (https://www.ed.ac.uk/usher/respire/health-literacy-asthma-malaysia/information-leaflets) before they gave written consent.

**Table 1:** Multicultural Malaysia and the health system^20^, ^21^

The health system	Multiracial and multilingual Malaysia
Malaysia has both public and private healthcare systems. The Government heavily funds the public health sector through taxation. The private sector is a 'fee for service' model often covered by insurance policies. The Government provides primary, secondary and tertiary care for the population. National Referral Centres provide specialized care and support the primary care service.^21^ In the public health sector, services are free with copayment ranging from MYR 1 (USD0.25) for outpatient services and MYR 3 (USD0.74) per day of admission.^22^	Malaysia is a multiracial country comprising three main ethnic groups: Malays, Chinese and Indians, each with their own culture and language.
	The national language, Malay, is used as the main medium of instruction in both primary and secondary national schools.
	English is learnt as a second language.
	At the primary school level, schools may also offer Mandarin and Tamil mediums of instruction.

Adult patients (aged ≥18 years) with physician‐diagnosed asthma on treatment from any of the four clinics, and with limited health literacy (defined as <33 on the validated Malay version of health literacy scale [HLS]^23^). We purposively recruited a maximum variation sample of participants based on demography (age, gender, ethnicity) and asthma control assessed using the asthma control questionnaire (ACQ).^24^ For those who agreed to participate, an initial interview was arranged at the most convenient clinic for the patient.

### 3.2 Data collection

Written informed consent was obtained before each activity. In Stage 1, the participant was invited for an initial in‐depth, semistructured interview. Using a topic guide (Supporting Information Appendix S1), the interviews started with a general discussion about personal experiences living with asthma. We then explored key areas of interest concerning participants' experiences of living with and managing asthma within and outside the home and interactions with healthcare professionals. H. S., a Malaysian family physician who designed the study in discussion with the wider research group, conducted all the interviews, assisted by an interpreter, T. P. Interviews were conducted in Malay, English, Tamil or Mandarin according to participants' preference, and they could move between languages if they so wished.

The photo‐taking activity (Stages 2 and 3) was offered to all participants at the end of the initial interview—there was no selection by the researcher at this stage. Those who agreed were given a 30‐min training on using a disposable camera and the ethics of photo‐taking (i.e., asking permission to take photographs of others). H. S. also provided a guide on using a disposable camera and how to take care of it. Each participant who agreed to photo‐taking was given a disposable camera containing 27‐picture film to complete the 2‐week activity. Participants were given a broad remit about photographs of interest (Table 2) and an infographic around photo‐taking rules (Supporting Information Appendix S2).

**Table 2:** Remit of the photo‐taking activity

Please take pictures that show:
(a) What is it like to live with asthma?
(b) What helps you, or what makes it more challenging to live with asthma?
(c) How do you learn about asthma, and where do you get information about it?

H. S. arranged for film development, digitalizing and printing photographs from returned cameras in preparation for Stage 3 interviews. At the Photovoice interview, H. S. displayed the photographs in hardcopy format and asked participants to choose the consent they wished to apply to each photograph. There were three options: (i) the photograph may be shared with the researcher and reproduced in publications with a caption of their choosing; (ii) the photograph (hardcopy form) may be shared with the researcher but not reproduced; and (iii) the photograph may not be shared with the researcher or reproduced (these were destroyed). This process allowed participants to understand the implications of allowing reproductions and to reflect and choose photographs they wished to share.

H. S. started the Photovoice interview using the photographs that the participant had agreed to share with the researcher, one at a time. Participants were encouraged to describe where, why the photographs were taken and what it meant to them particularly around the aspects of living with asthma. During the interview, participants were asked if there had been any situations they decided not to capture during the photo‐activity. H. S. encouraged participants to explain the reason behind these decisions (or why they decided not to share specific photographs with the researcher), though they were free not to explain their decision. The absence of these photographs challenged participants to reflect on pertinent—but hidden—issues such as social norms and practices. Participants were given space to add an explanation or accompanying text for each photograph. All interviews (in‐depth interviews and Photovoice interviews) were digitally audio‐recorded, using an encrypted audio‐recorder.

### 3.3 Data analysis

Recorded interviews were transcribed verbatim in the original language, checked for quality and accuracy and anonymized before translation into English using back‐to‐back translation methods.^22^ Where it was not possible to translate metaphors, idioms, or culturally specific expressions into English, the original language was maintained, and explanatory footnotes provided.^22^, ^25^ Table 3 describes steps for data analysis.

**Table 3:** Steps taken for data analysis^1^

1.	The analysis of the transcripts and the photographs were iteratively informed, but not restricted, by the health literacy framework by Sorensen.
2.	All the transcripts from the initial in‐depth interviews and photo‐interviews were uploaded in the computer‐aided qualitative analysis software (Nvivo 11).
3.	After reading and rereading to immerse in the data, H.S. conducted a preliminary analysis, which was then explored with I. Y./P. Y. L./S. S. G./H. P.
4.	H. S. then deductively coded the transcripts using the concerning asthma related‐health information; access, understand, appraise and apply.
5.	Each interview statement was coded into one of the broad themes of Sorensen's health literacy framework.
6.	We added additional categories to each theme to ensure we captured unique themes and conforming/nonconforming concepts against the initial assumptions about health literacy.
7.	Refinement, agreement of categories and subsequent themes were done in iterative discussion with the multidisciplinary research team (I. Y., P. Y. L., S. S. G., H. P.), providing diverse clinical, health system and social research backgrounds.
8.	The team identified and structured the themes presented in this article. We discussed preliminary analysis and captions accompanying the photographs with the participants enabling additional or discordant themes to emerge.

### 3.4 Patient and public involvement (PPI)

The research was discussed with PPI panels in Malaysia and the Asthma UK Centre for Applied Research (AUKCAR).^26^ They reviewed research documents (participant information sheet and consent forms, animated videos to overcome literacy problems, flyers and photo‐taking guidance) to improve readability and clarity. H. S. spoke with two PPI colleagues to learn more about how people with asthma view research and to discuss the topic guide. For example, one member suggested using common local terms to describe difficulty in breathing, such as *semput* for Malay participants. One of the PPI colleagues piloted the photo‐taking activity and made some practical suggestions (e.g., allowing 2‐week for the photo‐taking activity instead of 1).

### 3.5 Trustworthiness, reflexivity and power dynamics of research

As researchers, we significantly impact on how data are collected, shaped and analysed. Trustworthiness refers to the degree of confidence in data, interpretation and methods used to ensure the quality of a study.^27^ We adopted the Lincoln and Guba (1985, 1989) criteria to evaluate the credibility, transferability, dependability and conformability of our qualitative work.^28^, ^29^ We provide a detailed description of the strategies used in Supporting Information Appendix S3 with a summary in Table 4.

**Table 4:** **Summary of trustworthiness, reflexivity and power dynamics** ^27^, ^28^, ^29^

**Trustworthiness criteria**	**Research strategy**	**Techniques to ensure trustworthiness**
Credibility	1. Field notes/memo 2. Tape recorder 3. Auditing preliminary themes/analysis	1. Prolonged engagement 2. Member checking 3. Peer review 4. Reflexivity
This concept examines the correspondence between what participants say and how the researchers represent these viewpoints.		
Transferability	1. Data display 2. Simultaneous literature review	1. Sampling strategies 2. Thick descriptions of the context, setting and people studied.
This concept refers to the generalizability of inquiry. It asks how the study findings are generalized or applied to other individuals, groups, contexts or settings.		
Dependability	1. Field notes/memo 2. Tape recorder 3. Auditing preliminary themes/analysis	1. Audit trail of process logs 2. Peer‐review
This concept refers to the consistency of the data over time across researchers and methods.		
Conformability	1. Field notes/memo	1. Audit trail 2. Peer‐review 3. Member‐checking
This concept refers to the degree to which the respondents and conditions of the inquiry are determined and not of the interest and perspectives of the inquirer.		

Reflexivity and power dynamics	Techniques to address	
H. S. is a family physician who had more than 10 years of experience working in the Malaysian primary care setting.	Data analysis was discussed with a multidisciplinary research team, providing diverse insights into the study findings′ interpretation.	
Researchers from different settings: The RESPIRE Global Health Unit was funded by the National Institute of Health Research (NIHR), United Kingdom (UK).	The project was proposed, developed and conducted by the research team from Malaysia, ensuring understanding of local issues and resonance with the local sociocultural context.	
Researchers–participants relationship: Unequal power may make it challenging to elicit descriptions of social practices, explore personal issues such as identities and understanding how adults with limited health literacy live with asthma.	H. S. was introduced as a doctor and researcher by a local community leader, or via their doctors enhancing trust in sharing information with H. S. Also, at the photo interview, H. S. was known to participants from the previous interview. The photo activities shifted power to the participant as their choice of photos determined the topics of conversation.	

## 4 RESULTS

### 4.1 Description of data set

In total, 26 participants completed the initial interviews, 12 agreed to the photo‐taking training session, but only eight completed the photo discussion. Table 5 summarises the participants' characteristics. Reasons for nonparticipation for Stages 2 and 3 included time constraints and hospitalization for a severe exacerbation. Most (23/26) had uncontrolled asthma based on asthma control screening.

**Table 5:** Characteristics of participants

Profile	Subprofile	Initial interviews (*n* = 26)	Completed photo discussion (*n* = 8)
Sex	Male	8	2
	Female	18	6
Age category (years), mean age = 48.6	18–27	2	1
	28–37	7	3
	38–47	3	2
	48–57	4	2
	58–67	7	‐
	68–77	2	‐
	78–87	1	‐
Self‐assigned ethnicity	Malay	15	5
	Chinese	5	‐
	Indian	6	3
ACQ score^a^	Mean score	1.8	1.6
	Controlled, *n* (%)	3 (11.5)	2 (25)
	Uncontrolled, *n* (%)	23 (88.5)	6 (75)
Health literacy score	Mean^b^	23.7	25

Abbreviation: ACQ, asthma control questionnaire.

^a^ Score less than 0.75 = well‐controlled.

^b^ Mean less than 33 = limited health literacy.

### 4.2 Presentation of findings

**Figure 2:**
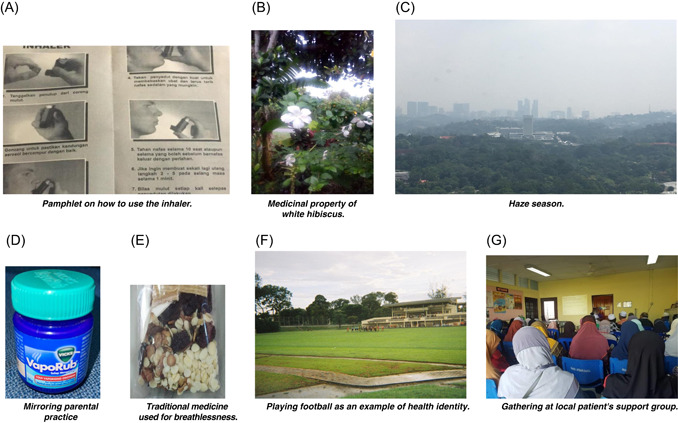
Photographs taken by the participants

We describe participants' understanding of asthma and how they decided on self‐management practices based on the four domains of health literacy: (i) access; (ii) understand; (iii) appraise; and (iv) apply.^1^ For each domain, we report the themes and subthemes that influenced participants' engagement with health service support for asthma self‐management. See Table 6 for a summary. Figure 2 are the photographs taken by the participants, which are related to many of the quotes.

**Table 6:** Summary of how participants understand asthma and decide on self‐management practices

Domains of health literacy^1^		Themes	Subthemes
1.	Access to information on asthma and self‐management	Formal source	Healthcare professionals as the primary source of knowledge on asthma
			Health pamphlet and talks improve asthma knowledge
		Informal source	Family members contribute to asthma knowledge
			Social media provides experiences of others
2.	Understanding information on asthma and self‐management	Patient factor	Use of national language on health information limits the understanding of those who speak their native language
		Healthcare professional factor	Communication skills of doctors affect understanding—verbal and nonverbal
3.	Appraisal of information on asthma and self‐management	Patient factor	Lack of appraisal strategies Experiential knowledge aid in evaluating the information on asthma
4.	Application of information on asthma and self‐management practices	Established self‐management practices are a mixture of medical narratives and social practices	Sociocultural norms and practice influence strategies to manage asthma symptoms
			Stigma may lead to a conflict of identity
			Social support embodiment of asthma identity

### 4.3 Access to information on asthma and self‐management

Participants described two sources of information regarding asthma and self‐management practices. The primary formal source of information was healthcare professionals. Participants considered the doctors had a duty to share information about asthma, its treatment and how to care for it at home. Doctors were perceived as health experts and trustworthy sources of information. Reliance on healthcare professional advice overcame participant's lack of confidence in finding and interpreting reliable information.

When the doctor explains, I listen to what he has to explain about asthma and the medications, because he's the expert. If I don't listen, I'll miss the things that I don't know, or I'm not sure of. I have to listen carefully [to the doctor] because I don't know where else to learn about these. I'm afraid, on my own, I may read the wrong information. I only depend on what the doctor told me. (63‐year‐old, Malay, woman)

Other sources of formal information were asthma‐related pamphlets and health talks. Participants described these sources as helping augment the knowledge shared by their doctors about asthma and how to care for it (Figure 2A).

I like to keep the pamphlets (showing a photograph) on how to use the inhaler from the health clinics. And sometimes, I'll go to the [health] talks. The content is similar to what the doctor told me, but, the pamphlets and the talks remind me of what I should always do to take care of my asthma, especially at home. It's like revision (chuckled). (31‐year‐old, Malay, woman)

Informal sources of information about asthma and self‐management practices also played an important role, especially recommendations from family members who had asthma. For instance, a participant described the pathophysiology of asthma based on what his sister shared with him.

I think I know why I get these attacks. My windpipe may be blocked because it swells up, probably when I'm allergic to something. So, the swelling closes the [wind]pipe, so my breathing becomes terrible. My sister told me about this; she has asthma too; way longer than I do. (33‐year‐old, Malay, man)

For those who had had asthma since childhood, parents were a source of knowledge about asthma and how to care for it (Figure 2B)

[This is] white hibiscus (showing a photograph of the flower). The flower has medicinal property. If I crushed it, it would give out sticky sap, and if I apply on the chest, it stops my cough. My mother used to do this since I was small. (41‐year‐old, Malay, woman)

Participants, especially those under 30 years, described reading about asthma on social media and were particularly interested in the experiences of others living with asthma. For example, one participant described using Facebook to read and find inspiration from others' experiences to live and care for her asthma.

I searched Facebook [to] see how people talk about their asthma. I like when they talk about what they cannot eat, see if it's the same issue [as] me. What they used when [their] breathing becomes not that great. I learnt from them. (26‐year‐old, Chinese, woman)

#### 4.3.1 Understanding information on asthma and self‐management

Participants described how both patient and healthcare professional factors influenced the process of understanding information once they had found relevant asthma/health information.

The language used was identified as a key issue. Participants pointed out that paper‐based information provided at the healthcare centres was mostly in Malay or English. Although most could read Malay, some struggled to understand these languages and described the need for services and/or asthma information packs in their native language to help them with their asthma care. One participant expressed her need for Mandarin‐version written information as she struggled to read Malay and had to ask for help with translation.

It [will] be good if there's Mandarin version of this [action] plan. It'll make my life easier. If the doctor speaks to me in Bahasa [Malay language], I can understand a little bit. But, reading this (the asthma action plan) in Bahasa, I don't quite understand. I have to ask for my husband's help to translate. (22‐year‐old, Chinese, woman)

Other participants used Internet tools such as ‘Google translate’ to interpret the information given to them. Even amongst participants who were able to read and speak colloquial Malay, some described difficulty understanding the formal Malay language used in asthma information. One participant, for instance, described the written asthma action plan as wordy and difficult to understand.

I can speak and read Malay [language], but that [asthma action] plan has too many words (chuckled). (78‐year‐old, Chinese, man)

Healthcare professionals were vital in facilitating an understanding of adherence to medication and the evidence‐based definition of asthma control. The lack of effective doctor–patient communication—verbal and nonverbal—was a central theme. For example, one participant described how doctors' lack of explanation made him question the treatment prescribed to him.

They (the doctors) will say do this, and that, but they don't even explain why. They said I have asthma and that I need to use the brown inhaler every day. But why? If I'm not sick, why should I use it? (66‐year‐old, Malay, man)

Some participants recalled their struggles to understand the conversation with their doctors and their asthma care plan because of medical jargon.

Doctors use many words I cannot understand, and I think those are English or is it science words? I'm not sure. Please use the language we ordinary people can understand. (41‐year‐old, Malay, woman)

Some participants observed nonreceptive body language from their healthcare professional, which deterred their efforts to clarify understanding information about asthma or the management plan.

When I came for follow up, the doctor barely looked at me. That's not really welcoming. I don't think that doctor is the type who wants to explain anything to me, so even if I don't understand anything, I'll just keep quiet. (61‐year‐old, Malay, woman)

#### 4.3.2 Appraisal of information on asthma and self‐management

Most participants did not explicitly evaluate information or check with health services to help them appraise information before applying it. When prompted, many participants stated that they had not thought of assessing the information given to them; they ‘just followed’. A participant unveiled a paternalistic culture she experienced, which led to such behaviour. She recalled,

I have been an asthma patient for the past 25 years. For many years, I take the medicines prescribed by the clinic. I do what I was told to. I don't question about right or wrong [on] anything the doctor tells me. I once asked [the doctor] about my breathing. I can't remember what exactly it was, but his words are deeply engraved in my head; ‘are you the doctor, or I am?’ I don't go to school, you see (trying to cover part of her face with her saree). I was embarrassed and [I] think, I made a fool of myself by questioning him. It was many years ago, I know they are nicer now, they really are, but since then, I never asked or questioned. So, I just follow. (62‐year‐old, Indian, woman)

Other than health information given by the healthcare professionals, information was typically assessed against their experience of living with asthma. For example, a participant highlighted how she weighed up the information about asthma triggers that she had found on social media and related this to her own experience before acting on the information (Figure 2C).

If I see posts on social media and everyone else agreed on it, then probably it's true. People said that the air‐conditioning system in the car makes people cough. It's the same with me, but, I still use it at times. Like during the haze, (showing a photograph), you cannot even see the sun, meaning it's awful. So, I must [switch] on the air‐con [air‐conditioning system] and rolled up my car's window. My asthma with haze is worse than with the air‐con. So, I have to choose the lesser evil. But one person is different from another, so you need to understand your own body. (38‐year‐old, Malay, woman)

#### 4.3.3 Application of knowledge on asthma and self‐management practices

Established self‐management practices were a mixture of medical narratives and social practices. Mixed‐use of prescribed medication from the health services and complementary and alternative medicine (CAM) was discussed in most interviews, particularly among people with uncontrolled asthma. CAM therapies and home remedies were widely used to counter symptoms. When participants reported beginning to feel any discomfort before the actual chest tightness or breathing difficulties, the ointment was often applied to the chest and throat. A participant described the use of ointment at the first sign of symptoms (Figure 2D), mirroring what her mother used during the early stages of an acute asthma exacerbation.

But my mom uses Vicks. So, since I was a kid, I do practice rubbing Vicks on the chest, like my mom used to do. That thing actually helps. Really. It really helps with the asthma, especially when you just about to feel it, the tightness. (31‐year‐old woman)

All but one of the participants brought at least one photograph of CAM that they used for asthma. Despite this, CAM use was rarely discussed in consultations, as they expected that the healthcare professionals would not be open to the idea. Close friends and family members were reported as actively recommending some of these therapies. For instance, one participant photographed the traditional herbal medications introduced by her neighbour (Figure 2E).

The dried crocodile meat, they cut into slices. My neighbour, she told me to boil it and drink, and your wheezing will disappear. I tried. Ah, I bought it. OK, I feel OK. It is just that I don't tell my doctor about this, you see. I don't think he will agree or he will believe this medicine. But I have to try, for my own sake. (54‐year‐old, Indian, woman)

Some who subscribed to the concept of Ying and Yang used warm water to counter asthma attacks. The hot and cold concept is widely understood in the Chinese culture, but it is also a concept accepted in Ayurvedic medicine in the Indian community. An Indian participant described and practised Ayurvedic medicine recommended by her mother‐in‐law to avoid being labelled as ‘sick’.

My mother‐in‐law practised Ayurvedic medicines. Ayurveda herbs and spices like black pepper are hot, she said, it's perfect for asthma. If it's hot, it can reduce the asthma attack. I always have the attack at night because it's cold. I'll straight chewed on the black pepper. Let it controlled my breathing, if that doesn't work then only, I'll take my inhaler. I don't need to go to the hospital that often anymore. Before this, she [mother‐in‐law] always asked why I'm always sick, not like other people (sighed). (37‐year‐old, Indian, woman)

Stigmatizing experiences were challenging and inevitably influenced self‐management decisions. Sports and physical activities such as playing football were identified as essential activities in embodying health identities, particularly among two young men in this study. Thus, for these participants, using an inhaler before a game or during a match demonstrated ‘weakness’ and invited unwanted social reactions. One participant shared a photograph of a football field where he used to play and said (Figure 2F):

When I feel like, I'm about to be breathless, and my breathing starts to be fast, I'll continue playing. Other people can't see it, and I don't want to ruin my reputation. Or else, no one wants me in their team if they know I'm sick. I'm a better football player than anyone on that field! Besides, anyone who is running, their breathing will be fast. So, it's normal. There's no big deal about it, and my breathing will be back to normal by the end of the game. (28‐year‐old, Malay, man)

In contrast, formal social support helped some participants adopt a strong sense of identity and a positive attitude towards guideline‐recommended care. One participant who joined a patient support group to improve physical health described how the weekly gathering had helped him embrace his identity and enabled him to empower others (Figure 2G).

I took this photo during one of our weekly gatherings. Usually, we started [with] a session by the doctor or the pharmacist. They gave talks about health, including asthma, that's where I know everything about how to take care of health. So, when other members [of the club] who don't have asthma listen to [the] talk about asthma, they know a little bit about it. The highlight of the day will be snack time! The club members [will] take the turn to organise this. Sometimes they get together and cook at their house, so we will enjoy some home‐cooked meal. It is during the mealtime that we chatted, we shared stories—good and bad. We feel belonged to something, and we are not ashamed of who we are; a person with asthma or diabetes or whatever it is. So, as a person with asthma, I actively find new asthma patients to join this club. Not just for them to know better about their illness but, to be among the people who understand what is it like to live with breathlessness. (56‐year‐old, Malay, man)

## 5 DISCUSSION

### 5.1 Summary of findings

Participants used formal and informal sources to access information about asthma and self‐management. Struggles with language (both ‘jargon’ and lack of translations) affected the understanding of written information, while good communication with a healthcare professional could help overcome this. Most participants did not explicitly describe strategies to evaluate or check the health information that they received. Participants in this study used experiential knowledge to help evaluate the information they received about asthma. Understanding asthma and self‐management practices were dominantly sociocultural norms or practices. Sense of identity was influenced by stigmatizing experiences and social support, which affected their decisions and self‐management choices.

### 5.2 Interpretation of results and comparison with the literature

#### 5.2.1 Communication and understanding how people process health information

In this study, people with limited health literacy relied heavily on others for information about their asthma and how to care for it, reflecting the findings of Edwards et al.,^30^ who concluded that people draw upon others' health literacy skills in search of health information. Our participants regarded healthcare professionals as legitimate sources of health information, as has been reported in other studies.^15^, ^31^, ^32^ Although healthcare professionals are aware of this responsibility, they may selectively provide health information to patients, that is those with poor disease control or those whom they perceive may understand the health information.^8^, ^31^, ^32^ Some patients may even wish to discuss CAM with their healthcare professionals but fear negative reactions.^33^ The healthcare professional's barriers to discussing CAM with patients include lack of a trusting relationship, personal disapproval of CAM and lack of evidence making a conversation about CAM uncomfortable.^34^

Our participants wanted to understand asthma and manage it but described challenges in their communications with the healthcare professionals. People with limited health literacy may not necessarily tackle communication barriers and may not use strategies such as establishing rapport and clarifying queries.^14^, ^32^, ^35^ How health information is communicated (e.g., using universal health literacy precautions in providing health information)^36^ is not only important to help patients understand their disease but is also crucial in dismantling paternalism in doctor–patient relationships.^37^, ^38^ Universal health literacy precautions include breaking down information and instructions into small steps, assessing comprehension through teach‐back cycle and use of visual aids.^36^, ^39^ Information tailored to the patient's health literacy needs is more likely to be translated into positive health behaviours.^37^, ^40^

Knowledge and illness experiences from family, close friends and stories shared via social media were trusted informal sources of information among participants in this study. It has been shown that people look to the Internet (especially social media) to find others with similar illness experiences and use these stories to learn new strategies or confirm that their health behaviour is appropriate.^32^, ^41^ The danger is that these experiences may not reflect accurate health information or recommended behaviours. A recent review that highlighted social media as a powerful channel for health communications similarly cautioned about unreliable content, lack of privacy of personal information.^42^ Others have suggested that superusers (users who write a large number of posts in online health communities) have the potential to reach a wide population and cost‐effectively support such communities by providing accurate information about asthma and awareness about guideline‐recommended practice.^43^

The lack of analytical skills to evaluate health information described by our participants could exacerbate the spread of false information and fearmongering against, for example, the use of preventer inhalers. Illness perceptions and personal health beliefs are grounded within social‐cultural norms and practices. Nonadherence, for example, has been linked to mismatches between patients' common‐sense interpretations of their long‐term illness and treatment and medical reasoning.^44^, ^45^ Acknowledging patient's views and empowering people with limited health literacy to understand their health and management has shown promising results.^46^ This has particular resonance in the context of the COVID pandemic as the world is addressing vaccination hesitancy, especially amongst some ethnic groups.^47^

#### 5.2.2 Social elements, identity and limited health literacy: The impact on asthma self‐management

Limited health literacy is not merely an individual trait. It is influenced by the characteristics of society and is a marker of multiple life circumstances and sociocultural challenges.^2^, ^17^, ^48^ It tends to affect vulnerable populations disproportionately, including people with lower educational attainment, people from ethnic minorities and those whose spoken language differs from the majority population. For example, our participants highlighted that health information in Malaysia was printed in Malay and English languages despite many people in this multilingual society speaking other languages, such as Mandarin and Tamil. This systematically hinders access to health information for some communities.

We found that language played a vital role in (inhibiting) knowledge exchange between the patients and their sources. Despite being a multilingual nation, interpreter services in Malaysian healthcare are scarce, echoing challenges reported in other healthcare services.^15^, ^49^ Typically, services depend on healthcare professionals to recognize and address the language problems,^8^, ^31^ commonly by using family members as translators. Internet‐based translations may not reflect the nuanced ethnic descriptions of illness.^14^, ^41^ Resonating with our findings, ethnic differences in describing common asthma symptoms in multilingual settings may affect doctor–patient communication, delaying diagnosis and timely care.^31^, ^50^

Adjusting to life with asthma can be challenging, and the participants with limited health literacy in our study used various strategies to negotiate how to live with asthma despite difficulties with understanding essential health information. Healthcare professionals may not be aware of these struggles and may lack the skills to address the patient's life issues and sociocultural challenges in disease management.^8^ Stigma directly impacted the lived experiences and decisions made by our participants. Goffman (1963) defines stigma as an ‘attribute that was deeply discrediting and reduced the bearer from a whole person to a tainted, discounted one’.^51^ In many societies, to be healthy is the norm to which ill‐health ought to be restored.^52^ Those with chronic illness (exemplified amongst our participants by the footballer and the daughter‐in‐law with asthma) struggled to meet this societal expectation and were assigned a sick identity, potentially affecting mental health.^53^ Our findings highlight the importance of not only focusing on the individual with asthma but on the broader social norms that provide the context to effective asthma self‐management. More recently, social movements championing health issues (such as Asthma Right Care Initiatives^54^) have significantly influenced health systems and are a force for societal change.^55^

#### 5.2.3 The role of adaptations in cross‐cultural research

In classic Photovoice methodology, the target community's voice‐making was conducted collectively.^18^ As participatory action research, it is bound to flexibility in terms of its conduct,^56^ particularly in overcoming sociocultural challenges (e.g., working with indigenous populations^57^). Adaptations include the use of individual interviews and community events at which participants' photographs were displayed on posters.^57^

We recognized the importance of promoting community voice, which is central to Photovoice. The photographs from this study have been used within the community, although not in a traditional local photo exhibition. In an age of global collaboration and the Internet, our definition of ‘community’ is not constrained by geography. Our ‘exhibition’ reached beyond the local community to influence national stakeholders in Malaysia (at a stakeholder meeting attended by one of the participants) and via publications and websites (https://www.ed.ac.uk/usher/respire/news/2021/world-asthma-day-photovoice-experiences-malaysia) to represent the global community of people living with asthma and limited health literacy. Although a 21st century adaptation of the Photovoice concept, it is our observation that this process has given ‘voice’ to this global community—and one we hope will increase as a number of colleagues in the NIHR Global Health Research Unit on Respiratory Health (RESPIRE) collaboration are planning similar Photovoice projects.

#### 5.2.4 Strengths and limitations

The Photovoice approach is conceived as participant‐led research, giving voice to people to communicate their experiences in their own way. We adapted this to a more researcher‐led approach, which has implications for the power balance between researcher and participants. For example, the researchers are clinicians, introducing a hierarchical power imbalance,^58^ which might deter coconstructions of knowledge with the participants and hinder understanding illness experiences in research interviews. We, therefore, put in place strategies to minimize the impacts on our research findings. Firstly, we used strategies described by Lincoln and Guba (see Table 4) to ensure the patients' voices are represented in the findings,^58^ which might deter coconstructions of knowledge with the participants and hinder understanding illness experiences in research interviews. We, therefore, put in place strategies to minimize the impacts on our research findings. Firstly, we used strategies described by Lincoln and Guba (see Table 4) to ensure the patients' voices are represented in the findings.^28^, ^29^ During interviews, we observed that photographs helped shift the power balance to the patient's experience as participants were reassured that the visualization helped clarify the experiences they were describing. We remained aware of the ethical and methodological strategies of Karnieli‐Miller et al.,^58^ for example, checking the accuracy of photograph captions and preliminary themes with the participants. Participant involvement is a relatively new concept in Malaysia that limited early‐stage involvement. Future studies could enable involvement with study conceptualization and design, further narrowing power differentials between researchers and participants.

The participants were recruited from multiple sites, purposively sampled to represent the varied sociodemographic backgrounds of the Malaysian population. Participation in Photovoice activities was offered to all participants with no selection by the researcher, though the topics covered in the interview may have influenced decisions to participate. We used robust approaches to ensure trustworthiness. We provided a participant video to supplement written information and help improve understanding of the research. Infographics explained the conduct of Photovoice and encouraged creativity in completing photo‐taking tasks.

The initial in‐depth interviews were representative of the intended range of demographic characteristics and asthma control, though only a minority of participants took part in the Photovoice phase, in part because of the time and commitment needed to complete this. As a result, we lacked older participants and those of Chinese ethnicity among those who completed the Photovoice interviews. In classic Photovoice methodology, we would only have recruited participants willing to commit to the photo‐taking. Our adapted approach with initial qualitative interviews meant we heard from a broader range of people enabling us to embed Photovoice insights in the broader context.

The use of a predetermined framework ensured a structured approach to developing our topic guides and informed our analysis. We remained aware, however, that this could restrict our data collection and interpretation of novel themes. We, therefore, explicitly looked for discordant views and unexpected themes. The Photovoice interviews enabled the participants to determine the topics for discussion, and because these progressed in parallel with the interviews, we could accommodate novel insights into the ongoing interviews. The presence (or lack) of photographs triggered discussion of issues important to the participants enabling them to set the agenda. Language barriers were overcome as participants used photographs to express their experiences, and the interviewer prompted discussion about persons, objects, or settings depicted in the photographs. Conversations about photographs they were unable to capture, triggered reflection on important—but hidden—concerns like mental health.^17^ The interviews and photographs provided a data source triangulation that enhanced understanding of health literacy experiences in real life and challenged us to think beyond the health system to how health literacy operates at the societal level. Interviews were conducted in the participant's spoken language to capture the cultural nuances, and we preserved this during the translation process.

### 5.3 Implications for policy, practice and research

**Figure 3:**
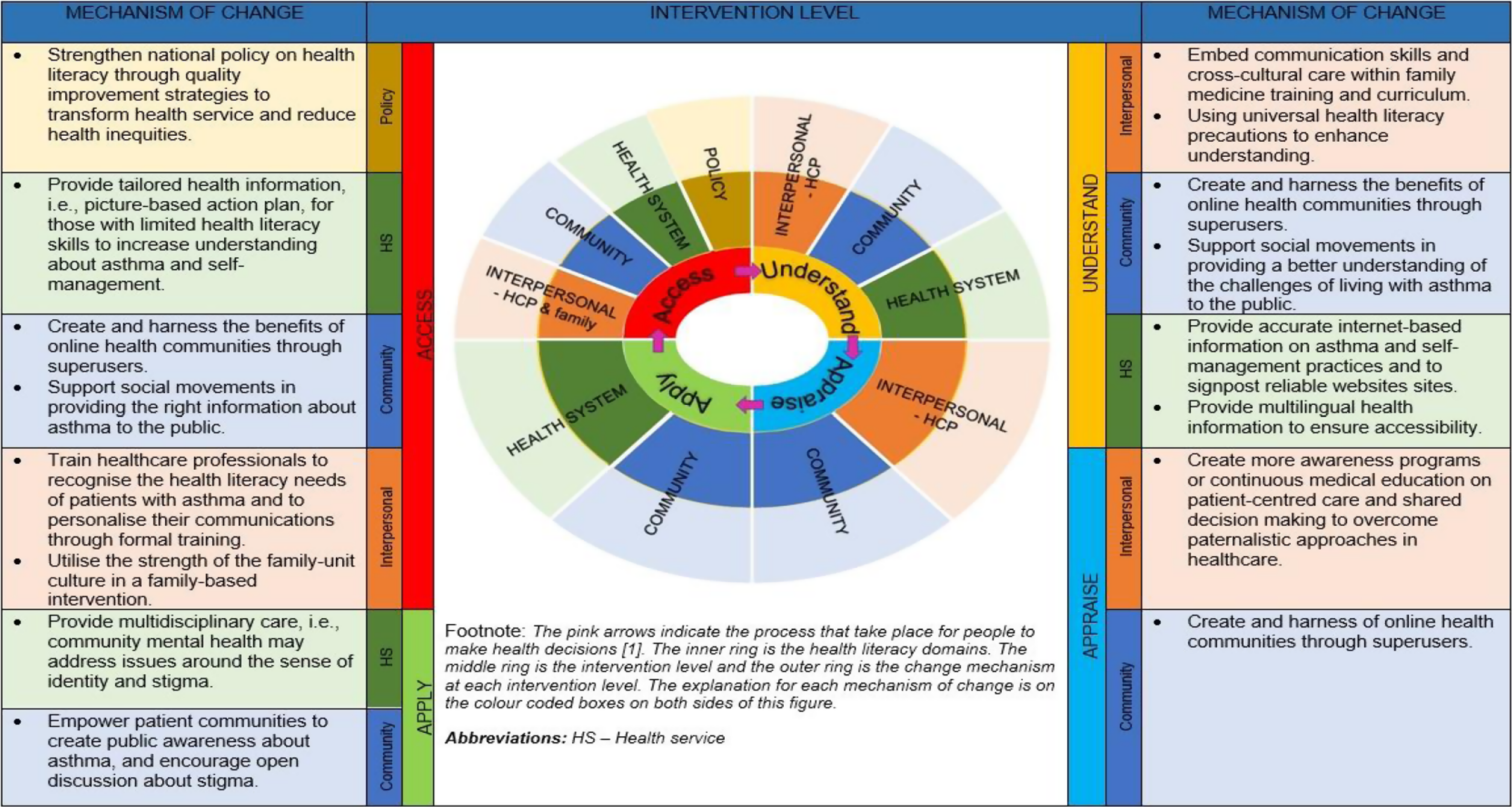
Change mechanisms that may be applied for interventions at different levels to improve understanding of asthma and self‐management practices

Tailored asthma care for patients' health literacy needs has a pivotal role in improving understanding of asthma and guideline‐recommended self‐management practices. Multilevel interventions (interpersonal, health system, community and policy) may be needed to achieve this. Based on our findings, we propose a framework for considering changes that might tackle health literacy issues in asthma care (Figure 3) and which potentially, may be adapted to other chronic conditions. Future research is needed to determine the feasibility and effectiveness of such multilevel interventions in low–middle‐income countries (LMICs) settings.

## 6 CONCLUSIONS

Health literacy and asthma self‐management remain a challenge in many healthcare settings, with particular challenges in multiethnic LMICs. We have described the experience of people with asthma in Malaysia based on an established health literacy framework. The photographs stimulated a unique insight into the challenges people with limited health literacy face in living with asthma and how they navigate the health system to care for their health. Multilevel, locally developed solutions that tailor health services, enable appropriate trusted information to support self‐management, and raising societal awareness about living with asthma may help people accept and adapt to the diagnosis, lessen stigma and cope with the demands of living with asthma.

## CONFLICT OF INTERESTS

The authors declare that there are no conflict of interests.

## AUTHOR CONTRIBUTIONS

Hani Salim, Ingrid Young, Ping Yein Lee, Sazlina Shariff‐Ghazali and Hilary Pinnock had full access to all the data and were involved in the interpretation of the data. Hani Salim wrote the initial draft of the paper with Hilary Pinnock to which all the authors contributed. Aziz Sheikh was Director of RESPIRE, led the securing and distribution of research funds and contributed to the development of the research protocol and overseeing the monitoring of its progress. Harish Nair commented critically on a draft of the manuscript. All authors approved the final draft.

## Supporting information

Supporting information.

Supporting information.

Supporting information.

## Data Availability

The datasets (texts) analysed during the current study are available in the Edinburgh DataShare repository, https://doi.org/10.7488/ds/2753. The photographs that support the findings of this study are available on request from the corresponding author. The data are not publicly available due to privacy or ethical restrictions.
