# Supplementary material for: Insights into how Malaysian adults with limited health literacy self‐manage and live with asthma: A Photovoice qualitative study
Source: Health Expect. 2021 Sep 12;25(1):163–76. doi: 10.1111/hex.13360 (PMC8849262; doi:10.1111/hex.13360)
Supplement: Supplementary file 1 — Supporting information. [file HEX-25-163-s001.docx]

**Appendix 1****: Topic guide**

| Topic | Question | Prompts |
| --- | --- | --- |
| **Asthma experience** | 1. Tell us about your experience as a person with asthma? | - The feeling of having asthma (stigmatization) - Understanding of asthma and its control. - Dealing with daily symptoms. - How long do you think your asthma will last? |
|  | 1. How does asthma affect your life? | - Daily activities? Work? Relationships - How you describe your condition to others? - Does what other opinion matter to you? |
|  | 1. How does life affect your asthma? |  |
|  | 1. Where do you find out about asthma? | - What do you have found out so far? - Source of information: Clinic? Family? Friends? Internet? |
| **Asthma management & control** | 1. Is your asthma well-controlled or troublesome? | - Why do you say so? - Why do these happen? - Who will get poor asthma control? - Do you think you are at risk of poor asthma control? - Can you give me an example of a time when you did not have good control of your asthma? What happened? What lead up to this? |
|  | 1. Can you tell me more about how your asthma being managed? (clinic or other alternatives) | - How do you think it should be managed? - If Alternative care: can you share more about it and why do you think you preferred this mode of care? - If hospital care: can you share how you being cared for by the healthcare system? Why do you think you preferred this mode of care? |
|  | 1. What is your opinion about the medical service provided to care for asthma? | - Healthcare personnel: Communication? - Facilities: accessibility? Cost? - Is routine care important? Why would some go for routine follow up? Would you engage in routine care & why? - How can it be improved? - Do you think inhalers works? Why is that so? |
| **Asthma self-management & support** | 1. Asthma isn’t always about doctors and clinics but about what you do every day. | - Can you tell me if you do anything in particular to help with your asthma? |
|  | 1. Have you heard about the asthma action plan? (Provide sample) | Participant knows about AAP   - Who educate you about it and what was discussed? How often are you reminded about using it? - How can this teaching session be improved? - Any benefit and problem using AAP? - If **no**, do you think there is a need to teach you on asthma action plan/self-managing asthma at home? - How can the doctor help you to do this? - Any other methods to facilitate you to use AAP in the future? |
|  | 1. What would help you to manage your own asthma? | - What format that you prefer? Example: Paper-based/Website/mobile apps/social media |
| **Closing** | 1. As a person with asthma, what are your hopes for the future? |  |
