# Supplementary figures and images for: Insights into how Malaysian adults with limited health literacy self‐manage and live with asthma: A Photovoice qualitative study
Source: Health Expect. 2021 Sep 12;25(1):163–76. doi: 10.1111/hex.13360 (PMC8849262; doi:10.1111/hex.13360)

**Appendix 2: Infographic pamphlet for photo-taking guide.**


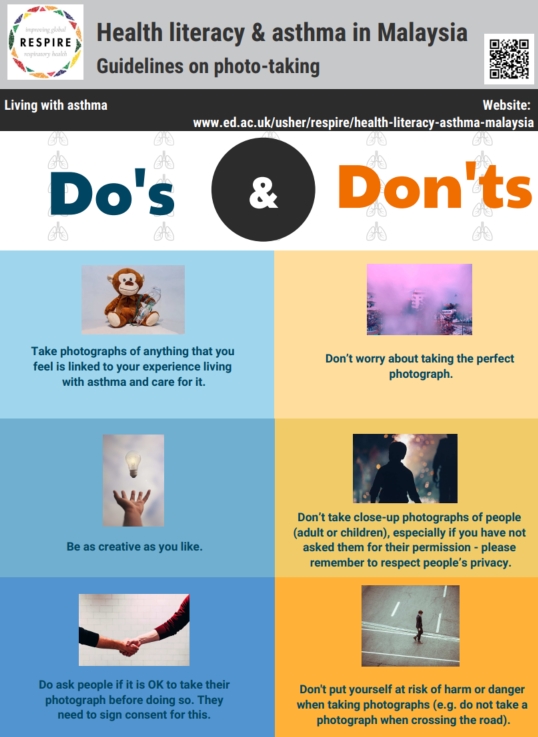

Supplement: Supplementary file 2 — Supporting information. [file HEX-25-163-s002.docx]
