# Supplementary material for: Insights into how Malaysian adults with limited health literacy self‐manage and live with asthma: A Photovoice qualitative study
Source: Health Expect. 2021 Sep 12;25(1):163–76. doi: 10.1111/hex.13360 (PMC8849262; doi:10.1111/hex.13360)
Supplement: Supplementary file 3 — Supporting information. [file HEX-25-163-s003.docx]

**Appendix 3: Descriptions of techniques to ensure trustworthiness.**

1. Prolonged engagement: Photovoice is a photo-interviewing method used to gain access to the community. The initial interview was critical for building rapport and trust with the participants. Overall, it took nearly eight months from the initial approach into the community to the final communication with interview participants. On-going communication took place through phone calls, messaging, and face-to-face meetings for recruitment, interviews, and preliminary analysis.
2. Member checking: During these meetings, we checked the accuracy of the descriptions accompanying the photographs and enquired about the preliminary themes of the interview - a practice known as member checking. Because we were more interested in the participants' preliminary analysis perspectives, we did not return to the original transcripts with them.
3. Peer review: The research was carried out in collaboration with researchers from various clinical and social research backgrounds. The multidisciplinary approach to analysis was enabled by the diverse experiences of cultural and social contexts of health practises. Several abstracts from this study were presented orally or as posters at seminars and conferences.
4. Sampling strategies: The study was first highlighted in February 2019 during a community health forum led by HS to provide information and raise awareness about asthma. The first participant was recruited through the community event, and the snowball effect began from there. Clinic members, particularly health professionals, also referred participants to the research booth. HS screened participants from a pool of volunteers, who had been screened for limited health literacy, and purposively sampled participants based on various demographic variables (age, gender, ethnicity) and asthma control profiles.
5. Thick descriptions of the context, setting, and people studied: Detailed descriptions about the participants, setting and context of the study were provided.
6. Audit trail of process logs: In a research journal, HS documented two aspects of the research: audit trails for decision-making and filed notes to capture reflections on the interviews and the research process, including various discussions with the broader research team.
